# Ethyl (2,5-dioxo-1-phenyl-2,3-dihydro-1*H*,5*H*-1-benzofuro[3,2-*d*]imidazo[1,2-*a*]pyrimidin-3-yl)acetate

**DOI:** 10.1107/S1600536810029521

**Published:** 2010-07-31

**Authors:** Shou-Heng Deng, Feng-Jun Cao, Xiao-Jun Cai, Fang Li, Ping Chen

**Affiliations:** a Center of Oncology, People’s Hospital of Hubei Medical University, Shiyan Hubei 442000, People’s Republic of China

## Abstract

In the title compound, C_22_H_17_N_3_O_5_, synthesized *via* the aza-Wittig reaction of ethyl 3-(phenyl­imino­methyl­ene­amino)­benzofuran-2-carboxyl­ate, benzene isocyanate and diethyl 2-amino­succinate, the imidazo[1,2-*a*]benzo[4,5]furo[2,3-*d*]pyrim­idine ring system is essentially planar (r.m.s. deviation for all 16 non-H atoms = 0.020 Å). The phenyl ring is twisted with respect to this ring system, making a dihedral angle of 54.23 (4)°. The crystal packing is stabilized by weak inter­molecular C—H⋯O inter­actions.

## Related literature

The title compound may be used as a precursor for obtaining bioactive mol­ecules, see: Bellarosa *et al.* (1996 ▶). For the biological activity of benzofuropyrimidine derivatives, see: Moneam *et al.* (2004 ▶); Bodke *et al.* (2003 ▶); Palacios *et al.* (2007 ▶); Duval *et al.* (2005 ▶); Teimouria *et al.* (2006 ▶). For the crystal structures of other fused pyrimidinone derivatives, see: Hu *et al.* (2005 ▶, 2006 ▶, 2007 ▶, 2008 ▶).
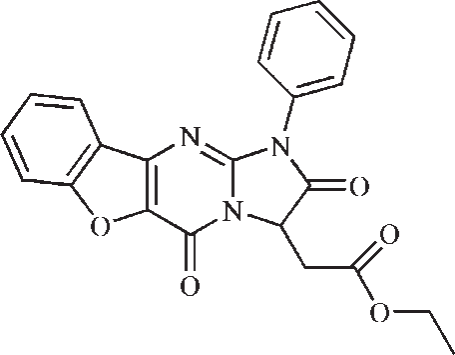


## Experimental

### 

#### Crystal data

C_22_H_17_N_3_O_5_
*M* *_r_* = 403.39
Triclinic, 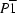

*a* = 8.5418 (12) Å
*b* = 8.6553 (12) Å
*c* = 14.519 (2) Å
α = 86.642 (2)°
β = 82.873 (2)°
γ = 62.619 (2)°
*V* = 945.8 (2) Å^3^
*Z* = 2
Mo *K*α radiation
μ = 0.10 mm^−1^
*T* = 292 K
0.30 × 0.20 × 0.10 mm

#### Data collection

Bruker SMART 4K CCD area-detector diffractometer
Absorption correction: multi-scan (*SADABS*; Sheldrick, 2003 ▶) *T*_min_ = 0.970, *T*_max_ = 0.990
5574 measured reflections
3663 independent reflections
2844 reflections with *I* > 2σ(*I*)
*R*_int_ = 0.084

#### Refinement

*R*[*F* ^2^ > 2σ(*F* ^2^)] = 0.045
*wR*(*F* ^2^) = 0.129
*S* = 1.06
3663 reflections
272 parameters
H-atom parameters constrained
Δρ_max_ = 0.25 e Å^−3^
Δρ_min_ = −0.35 e Å^−3^

### 

Data collection: *SMART* (Bruker, 2001 ▶); cell refinement: *SAINT-Plus* (Bruker, 2001 ▶); data reduction: *SAINT-Plus*; program(s) used to solve structure: *SHELXS97* (Sheldrick, 2008 ▶); program(s) used to refine structure: *SHELXL97* (Sheldrick, 2008 ▶); molecular graphics: *PLATON* (Spek, 2009 ▶); software used to prepare material for publication: *SHELXTL* (Sheldrick, 2008 ▶).

## Supplementary Material

Crystal structure: contains datablocks I, global. DOI: 10.1107/S1600536810029521/bt5306sup1.cif

Structure factors: contains datablocks I. DOI: 10.1107/S1600536810029521/bt5306Isup2.hkl

Additional supplementary materials:  crystallographic information; 3D view; checkCIF report

## Figures and Tables

**Table 1:** Hydrogen-bond geometry (Å, °)

*D*—H⋯*A*	*D*—H	H⋯*A*	*D*⋯*A*	*D*—H⋯*A*
C2—H2⋯O5^i^	0.93	2.56	3.442 (2)	159
C3—H3⋯O4^ii^	0.93	2.58	3.305 (2)	135
C5—H5⋯O2^iii^	0.93	2.46	3.132 (2)	129
C15—H15⋯O3^iv^	0.93	2.53	3.434 (2)	163
C19—H19*A*⋯O2	0.97	2.52	3.137 (2)	122

Symmetry codes: (i) 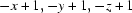; (ii) 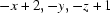; (iii) 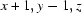; (iv) 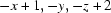.

**Fig. 1.**
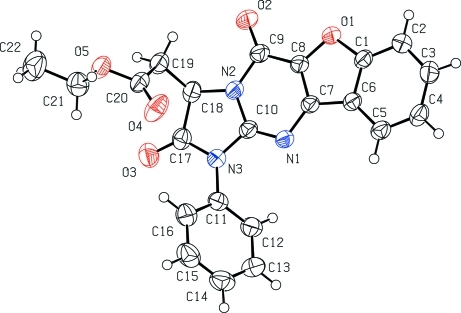
The molecular structure of the title compound, showing the atom-labeling scheme. Displacement ellipsoids are at the 50% probability level.

## supplementary crystallographic information

### Comment

The derivatives of benzofuropyrimidine are of great importance because of their remarkable biological properties. Some of them have shown good analgesic, anti-inflammatory and antimicrobial activities (Moneam *et al.*, 2004 and Bodke *et al.*, 2003). On the other hand, heterocycles containing an imidazolone nucleus also exhibit various biological activities. Several of them have shown good antibacterial, antifungal activities or are used as leukotriene B4 receptor antagonist and potassium channel openers (Palacios *et al.*, 2007 and Duval *et al.*, 2005, Teimouria *et al.*, 2006). The introduction of an imidazolone ring to the benzofuro[3,2-*d*]pyrimidin-4(3*H*)-one system is expected to influence the biological activities significantly. As a part of our ongoing investigations on the preparation of derivatives of heterocyclic compounds (Hu *et al.*, 2005, 2006, 2007, 2008), we have synthesized and structurally characterized  the title compound, and here we   report its  crystal structure (Fig. 1).

In the crystal structure of the title compound, all ring atoms of imidazo[1,2-*a*]benzo[4,5]furo [2,3-*d*]pyrimidine system are essentially coplanar, with maximum deviations -0.039 (3)Å and 0.057 (1)Å for O3 and N2, respectively. The phenyl (C11—C16) ring is twisted with respect to it, making dihedral angles of 54.23 (4)°. The structure is mainly stabilized by weak C—H···O   interactions.

### Experimental

The title compound was obtained in excellent yield *via* aza-Wittig reaction. Crystals suitable for single-crystal X-ray diffraction were obtained by recrystallization from a mixed solvent of ethanol and dichloromethane (1:2 *v*/*v*) at room temperature.

### Refinement

All H-atoms were found in a difference map but positioned with idealized geometry and refined with *U*_iso_(H)= 1.5*U*_eq_(C) for methyl H atoms and *U*_iso_(H) =1.2*U*_eq_(C) for all other H atoms using a riding model with C—H ranging from 0.93Å to 0.97Å.

### Crystal data

**Table:**

C_22_H_17_N_3_O_5_	*Z* = 2
*M**_r_* = 403.39	*F*(000) = 420
Triclinic, *P*1	*D*_x_ = 1.416 Mg m^−^^3^
Hall symbol: -P 1	Mo *K*α radiation, λ = 0.71073 Å
*a* = 8.5418 (12) Å	Cell parameters from 2477 reflections
*b* = 8.6553 (12) Å	θ = 6.0–25.0°
*c* = 14.519 (2) Å	µ = 0.10 mm^−^^1^
α = 86.642 (2)°	*T* = 292 K
β = 82.873 (2)°	Block, colourless
γ = 62.619 (2)°	0.30 × 0.20 × 0.10 mm
*V* = 945.8 (2) Å^3^	

### Data collection

**Table:**

Bruker SMART 4K CCD area-detector diffractometer	3663 independent reflections
Radiation source: fine-focus sealed tube	2844 reflections with *I* > 2σ(*I*)
graphite	*R*_int_ = 0.084
φ and ω scans	θ_max_ = 26.0°, θ_min_ = 2.7°
Absorption correction: multi-scan (*SADABS*; Sheldrick, 2003)	*h* = −10→10
*T*_min_ = 0.970, *T*_max_ = 0.990	*k* = −5→10
5574 measured reflections	*l* = −17→17

### Refinement

**Table:**

Refinement on *F*^2^	Primary atom site location: structure-invariant direct methods
Least-squares matrix: full	Secondary atom site location: difference Fourier map
*R*[*F*^2^ > 2σ(*F*^2^)] = 0.045	Hydrogen site location: inferred from neighbouring sites
*wR*(*F*^2^) = 0.129	H-atom parameters constrained
*S* = 1.06	*w* = 1/[σ^2^(*F*_o_^2^) + (0.0729*P*)^2^ + 0.002*P*] where *P* = (*F*_o_^2^ + 2*F*_c_^2^)/3
3663 reflections	(Δ/σ)_max_ = 0.003
272 parameters	Δρ_max_ = 0.25 e Å^−^^3^
0 restraints	Δρ_min_ = −0.35 e Å^−^^3^

### Special details

**Table:**

Geometry. All e.s.d.'s (except the e.s.d. in the dihedral angle between two l.s. planes) are estimated using the full covariance matrix. The cell e.s.d.'s are taken into account individually in the estimation of e.s.d.'s in distances, angles and torsion angles; correlations between e.s.d.'s in cell parameters are only used when they are defined by crystal symmetry. An approximate (isotropic) treatment of cell e.s.d.'s is used for estimating e.s.d.'s involving l.s. planes.
Refinement. Refinement of *F*^2^ against ALL reflections. The weighted *R*-factor *wR* and goodness of fit *S* are based on *F*^2^, conventional *R*-factors *R* are based on *F*, with *F* set to zero for negative *F*^2^. The threshold expression of *F*^2^ > σ(*F*^2^) is used only for calculating *R*-factors(gt) *etc*. and is not relevant to the choice of reflections for refinement. *R*-factors based on *F*^2^ are statistically about twice as large as those based on *F*, and *R*- factors based on ALL data will be even larger.

### Fractional atomic coordinates and isotropic or equivalent isotropic displacement parameters (Å^2^)

**Table:**

	*x*	*y*	*z*	*U*_iso_*/*U*_eq_	
C1	0.8363 (2)	0.04672 (19)	0.37332 (10)	0.0385 (4)	
C2	0.9489 (2)	0.0189 (2)	0.29234 (11)	0.0477 (4)	
H2	0.9210	0.0987	0.2437	0.057*	
C3	1.1056 (2)	−0.1346 (2)	0.28769 (11)	0.0492 (4)	
H3	1.1857	−0.1587	0.2345	0.059*	
C4	1.1467 (2)	−0.2539 (2)	0.36064 (11)	0.0461 (4)	
H4	1.2535	−0.3556	0.3551	0.055*	
C5	1.0323 (2)	−0.2243 (2)	0.44088 (11)	0.0414 (4)	
H5	1.0602	−0.3045	0.4893	0.050*	
C6	0.8732 (2)	−0.07023 (19)	0.44725 (10)	0.0361 (3)	
C7	0.72423 (19)	0.00914 (18)	0.51685 (10)	0.0352 (3)	
C8	0.6108 (2)	0.16444 (19)	0.48043 (10)	0.0382 (4)	
C9	0.4462 (2)	0.2830 (2)	0.52864 (11)	0.0422 (4)	
C10	0.5479 (2)	0.05584 (19)	0.64773 (10)	0.0366 (4)	
C11	0.5896 (2)	−0.1152 (2)	0.79340 (10)	0.0411 (4)	
C12	0.6649 (2)	−0.2842 (2)	0.76226 (12)	0.0493 (4)	
H12	0.6413	−0.3092	0.7056	0.059*	
C13	0.7760 (2)	−0.4161 (2)	0.81653 (13)	0.0547 (5)	
H13	0.8279	−0.5307	0.7962	0.066*	
C14	0.8108 (2)	−0.3794 (3)	0.90068 (13)	0.0580 (5)	
H14	0.8876	−0.4687	0.9362	0.070*	
C15	0.7316 (2)	−0.2107 (3)	0.93184 (13)	0.0609 (5)	
H15	0.7522	−0.1864	0.9894	0.073*	
C16	0.6215 (2)	−0.0769 (2)	0.87790 (11)	0.0530 (4)	
H16	0.5696	0.0376	0.8984	0.064*	
C17	0.3210 (2)	0.1644 (2)	0.76449 (11)	0.0430 (4)	
C18	0.2735 (2)	0.2986 (2)	0.68624 (10)	0.0422 (4)	
H18	0.1697	0.3053	0.6606	0.051*	
C19	0.2319 (2)	0.4798 (2)	0.71761 (11)	0.0473 (4)	
H19A	0.2548	0.5430	0.6646	0.057*	
H19B	0.1068	0.5419	0.7397	0.057*	
C20	0.3383 (2)	0.4779 (2)	0.79307 (11)	0.0424 (4)	
C21	0.3358 (2)	0.6322 (2)	0.92299 (12)	0.0550 (5)	
H21A	0.4454	0.6372	0.9012	0.066*	
H21B	0.3634	0.5308	0.9626	0.066*	
C22	0.2109 (3)	0.7929 (3)	0.97571 (15)	0.0814 (7)	
H22A	0.1900	0.8929	0.9371	0.122*	
H22B	0.2614	0.8000	1.0298	0.122*	
H22C	0.1007	0.7893	0.9943	0.122*	
N1	0.69428 (17)	−0.05275 (16)	0.60320 (8)	0.0381 (3)	
N2	0.42881 (16)	0.21602 (16)	0.61779 (8)	0.0392 (3)	
N3	0.48245 (17)	0.02592 (16)	0.73590 (8)	0.0413 (3)	
O1	0.67400 (14)	0.19234 (13)	0.39205 (7)	0.0442 (3)	
O2	0.33196 (16)	0.42088 (15)	0.50277 (8)	0.0599 (4)	
O3	0.23141 (16)	0.17923 (16)	0.83798 (8)	0.0561 (3)	
O4	0.48112 (15)	0.36241 (16)	0.80601 (10)	0.0646 (4)	
O5	0.25135 (14)	0.62047 (14)	0.84498 (8)	0.0501 (3)	

### Atomic displacement parameters (Å^2^)

**Table:**

	*U*^11^	*U*^22^	*U*^33^	*U*^12^	*U*^13^	*U*^23^
C1	0.0384 (8)	0.0302 (8)	0.0392 (8)	−0.0076 (7)	−0.0086 (6)	−0.0031 (6)
C2	0.0559 (10)	0.0411 (9)	0.0391 (9)	−0.0164 (8)	−0.0047 (7)	0.0019 (7)
C3	0.0470 (10)	0.0478 (10)	0.0451 (9)	−0.0156 (8)	0.0005 (8)	−0.0065 (8)
C4	0.0367 (8)	0.0387 (9)	0.0510 (10)	−0.0056 (7)	−0.0073 (7)	−0.0087 (8)
C5	0.0429 (9)	0.0336 (8)	0.0421 (9)	−0.0105 (7)	−0.0132 (7)	−0.0004 (7)
C6	0.0400 (8)	0.0310 (8)	0.0349 (8)	−0.0122 (7)	−0.0112 (6)	−0.0021 (6)
C7	0.0397 (8)	0.0298 (8)	0.0336 (8)	−0.0118 (7)	−0.0115 (6)	−0.0002 (6)
C8	0.0434 (9)	0.0325 (8)	0.0328 (8)	−0.0113 (7)	−0.0088 (6)	0.0006 (6)
C9	0.0455 (9)	0.0336 (8)	0.0394 (9)	−0.0096 (7)	−0.0123 (7)	0.0011 (7)
C10	0.0425 (8)	0.0290 (8)	0.0364 (8)	−0.0131 (7)	−0.0097 (6)	−0.0016 (6)
C11	0.0463 (9)	0.0405 (9)	0.0369 (8)	−0.0206 (8)	−0.0052 (7)	0.0044 (7)
C12	0.0645 (11)	0.0440 (10)	0.0416 (9)	−0.0260 (9)	−0.0097 (8)	0.0034 (7)
C13	0.0581 (11)	0.0401 (10)	0.0590 (11)	−0.0171 (9)	−0.0069 (9)	0.0066 (8)
C14	0.0498 (10)	0.0613 (12)	0.0549 (11)	−0.0191 (9)	−0.0122 (8)	0.0176 (9)
C15	0.0632 (12)	0.0756 (14)	0.0420 (10)	−0.0287 (11)	−0.0136 (8)	0.0033 (9)
C16	0.0632 (11)	0.0542 (11)	0.0425 (9)	−0.0266 (9)	−0.0082 (8)	−0.0031 (8)
C17	0.0439 (9)	0.0425 (9)	0.0425 (9)	−0.0188 (8)	−0.0050 (7)	−0.0072 (7)
C18	0.0379 (8)	0.0406 (9)	0.0421 (9)	−0.0119 (7)	−0.0051 (7)	−0.0076 (7)
C19	0.0458 (9)	0.0385 (9)	0.0464 (9)	−0.0088 (8)	−0.0058 (7)	−0.0060 (7)
C20	0.0364 (9)	0.0330 (8)	0.0502 (9)	−0.0098 (7)	−0.0004 (7)	−0.0049 (7)
C21	0.0577 (11)	0.0603 (12)	0.0495 (10)	−0.0277 (10)	−0.0094 (8)	−0.0048 (9)
C22	0.0964 (17)	0.0683 (14)	0.0747 (15)	−0.0303 (13)	−0.0108 (12)	−0.0264 (12)
N1	0.0424 (7)	0.0304 (7)	0.0353 (7)	−0.0105 (6)	−0.0085 (6)	0.0006 (5)
N2	0.0403 (7)	0.0319 (7)	0.0368 (7)	−0.0083 (6)	−0.0064 (6)	−0.0020 (5)
N3	0.0468 (8)	0.0347 (7)	0.0371 (7)	−0.0143 (6)	−0.0036 (6)	−0.0006 (5)
O1	0.0457 (6)	0.0329 (6)	0.0374 (6)	−0.0038 (5)	−0.0062 (5)	0.0032 (5)
O2	0.0555 (7)	0.0413 (7)	0.0512 (7)	0.0054 (6)	−0.0110 (6)	0.0065 (6)
O3	0.0540 (7)	0.0614 (8)	0.0462 (7)	−0.0224 (6)	0.0045 (6)	−0.0055 (6)
O4	0.0422 (7)	0.0484 (8)	0.0882 (10)	−0.0036 (6)	−0.0173 (6)	−0.0173 (7)
O5	0.0492 (7)	0.0386 (7)	0.0494 (7)	−0.0065 (5)	−0.0104 (5)	−0.0098 (5)

### Geometric parameters (Å, °)

**Table:**

C1—C2	1.380 (2)	C13—C14	1.380 (2)
C1—O1	1.3871 (17)	C13—H13	0.9300
C1—C6	1.393 (2)	C14—C15	1.374 (3)
C2—C3	1.384 (2)	C14—H14	0.9300
C2—H2	0.9300	C15—C16	1.383 (2)
C3—C4	1.394 (2)	C15—H15	0.9300
C3—H3	0.9300	C16—H16	0.9300
C4—C5	1.378 (2)	C17—O3	1.2096 (18)
C4—H4	0.9300	C17—N3	1.381 (2)
C5—C6	1.396 (2)	C17—C18	1.527 (2)
C5—H5	0.9300	C18—N2	1.4638 (18)
C6—C7	1.439 (2)	C18—C19	1.524 (2)
C7—C8	1.368 (2)	C18—H18	0.9800
C7—N1	1.3756 (18)	C19—C20	1.502 (2)
C8—O1	1.3811 (18)	C19—H19A	0.9700
C8—C9	1.426 (2)	C19—H19B	0.9700
C9—O2	1.2183 (18)	C20—O4	1.1984 (18)
C9—N2	1.406 (2)	C20—O5	1.3303 (18)
C10—N1	1.2863 (19)	C21—O5	1.4482 (19)
C10—N2	1.3738 (19)	C21—C22	1.488 (2)
C10—N3	1.3910 (19)	C21—H21A	0.9700
C11—C12	1.378 (2)	C21—H21B	0.9700
C11—C16	1.380 (2)	C22—H22A	0.9600
C11—N3	1.438 (2)	C22—H22B	0.9600
C12—C13	1.381 (2)	C22—H22C	0.9600
C12—H12	0.9300		
			
C2—C1—O1	125.12 (14)	C16—C15—H15	119.9
C2—C1—C6	123.52 (15)	C11—C16—C15	119.29 (17)
O1—C1—C6	111.37 (13)	C11—C16—H16	120.4
C1—C2—C3	116.09 (16)	C15—C16—H16	120.4
C1—C2—H2	122.0	O3—C17—N3	127.00 (16)
C3—C2—H2	122.0	O3—C17—C18	125.28 (15)
C2—C3—C4	121.75 (15)	N3—C17—C18	107.72 (13)
C2—C3—H3	119.1	N2—C18—C19	115.56 (13)
C4—C3—H3	119.1	N2—C18—C17	101.49 (12)
C5—C4—C3	121.36 (15)	C19—C18—C17	113.30 (12)
C5—C4—H4	119.3	N2—C18—H18	108.7
C3—C4—H4	119.3	C19—C18—H18	108.7
C4—C5—C6	117.96 (15)	C17—C18—H18	108.7
C4—C5—H5	121.0	C20—C19—C18	113.46 (13)
C6—C5—H5	121.0	C20—C19—H19A	108.9
C1—C6—C5	119.32 (14)	C18—C19—H19A	108.9
C1—C6—C7	105.44 (13)	C20—C19—H19B	108.9
C5—C6—C7	135.23 (14)	C18—C19—H19B	108.9
C8—C7—N1	124.78 (14)	H19A—C19—H19B	107.7
C8—C7—C6	106.21 (13)	O4—C20—O5	124.28 (15)
N1—C7—C6	129.01 (13)	O4—C20—C19	124.77 (14)
C7—C8—O1	112.30 (13)	O5—C20—C19	110.94 (13)
C7—C8—C9	123.50 (14)	O5—C21—C22	107.98 (15)
O1—C8—C9	124.20 (13)	O5—C21—H21A	110.1
O2—C9—N2	121.73 (15)	C22—C21—H21A	110.1
O2—C9—C8	129.66 (16)	O5—C21—H21B	110.1
N2—C9—C8	108.61 (13)	C22—C21—H21B	110.1
N1—C10—N2	127.32 (14)	H21A—C21—H21B	108.4
N1—C10—N3	124.27 (14)	C21—C22—H22A	109.5
N2—C10—N3	108.40 (13)	C21—C22—H22B	109.5
C12—C11—C16	121.05 (16)	H22A—C22—H22B	109.5
C12—C11—N3	120.22 (13)	C21—C22—H22C	109.5
C16—C11—N3	118.67 (15)	H22A—C22—H22C	109.5
C11—C12—C13	118.91 (15)	H22B—C22—H22C	109.5
C11—C12—H12	120.5	C10—N1—C7	111.84 (13)
C13—C12—H12	120.5	C10—N2—C9	123.79 (13)
C14—C13—C12	120.62 (17)	C10—N2—C18	111.50 (12)
C14—C13—H13	119.7	C9—N2—C18	124.39 (13)
C12—C13—H13	119.7	C17—N3—C10	110.86 (13)
C15—C14—C13	119.84 (17)	C17—N3—C11	125.99 (13)
C15—C14—H14	120.1	C10—N3—C11	122.06 (13)
C13—C14—H14	120.1	C8—O1—C1	104.69 (11)
C14—C15—C16	120.26 (17)	C20—O5—C21	116.76 (13)
C14—C15—H15	119.9		
			
O1—C1—C2—C3	179.50 (14)	C18—C19—C20—O4	26.2 (2)
C6—C1—C2—C3	−0.4 (2)	C18—C19—C20—O5	−152.67 (14)
C1—C2—C3—C4	0.1 (2)	N2—C10—N1—C7	0.0 (2)
C2—C3—C4—C5	0.1 (3)	N3—C10—N1—C7	178.67 (13)
C3—C4—C5—C6	−0.2 (2)	C8—C7—N1—C10	−2.0 (2)
C2—C1—C6—C5	0.4 (2)	C6—C7—N1—C10	178.43 (14)
O1—C1—C6—C5	−179.53 (13)	N1—C10—N2—C9	3.6 (2)
C2—C1—C6—C7	179.39 (14)	N3—C10—N2—C9	−175.22 (13)
O1—C1—C6—C7	−0.51 (16)	N1—C10—N2—C18	177.39 (14)
C4—C5—C6—C1	−0.1 (2)	N3—C10—N2—C18	−1.42 (16)
C4—C5—C6—C7	−178.74 (15)	O2—C9—N2—C10	175.24 (15)
C1—C6—C7—C8	0.13 (16)	C8—C9—N2—C10	−4.5 (2)
C5—C6—C7—C8	178.92 (17)	O2—C9—N2—C18	2.2 (2)
C1—C6—C7—N1	179.78 (14)	C8—C9—N2—C18	−177.54 (13)
C5—C6—C7—N1	−1.4 (3)	C19—C18—N2—C10	124.87 (14)
N1—C7—C8—O1	−179.38 (12)	C17—C18—N2—C10	1.87 (15)
C6—C7—C8—O1	0.29 (17)	C19—C18—N2—C9	−61.38 (19)
N1—C7—C8—C9	0.5 (2)	C17—C18—N2—C9	175.62 (13)
C6—C7—C8—C9	−179.86 (14)	O3—C17—N3—C10	−178.92 (15)
C7—C8—C9—O2	−177.07 (16)	C18—C17—N3—C10	0.95 (16)
O1—C8—C9—O2	2.8 (3)	O3—C17—N3—C11	−10.7 (3)
C7—C8—C9—N2	2.7 (2)	C18—C17—N3—C11	169.13 (13)
O1—C8—C9—N2	−177.49 (13)	N1—C10—N3—C17	−178.61 (14)
C16—C11—C12—C13	−1.0 (3)	N2—C10—N3—C17	0.25 (17)
N3—C11—C12—C13	176.04 (15)	N1—C10—N3—C11	12.7 (2)
C11—C12—C13—C14	0.2 (3)	N2—C10—N3—C11	−168.47 (13)
C12—C13—C14—C15	1.3 (3)	C12—C11—N3—C17	134.92 (17)
C13—C14—C15—C16	−2.0 (3)	C16—C11—N3—C17	−48.0 (2)
C12—C11—C16—C15	0.3 (3)	C12—C11—N3—C10	−58.1 (2)
N3—C11—C16—C15	−176.76 (15)	C16—C11—N3—C10	118.95 (17)
C14—C15—C16—C11	1.2 (3)	C7—C8—O1—C1	−0.59 (16)
O3—C17—C18—N2	178.21 (15)	C9—C8—O1—C1	179.56 (14)
N3—C17—C18—N2	−1.66 (15)	C2—C1—O1—C8	−179.22 (14)
O3—C17—C18—C19	53.7 (2)	C6—C1—O1—C8	0.68 (16)
N3—C17—C18—C19	−126.20 (14)	O4—C20—O5—C21	−1.4 (2)
N2—C18—C19—C20	−83.04 (17)	C19—C20—O5—C21	177.50 (14)
C17—C18—C19—C20	33.47 (19)	C22—C21—O5—C20	−175.36 (16)

### Hydrogen-bond geometry (Å, °)

**Table:**

*D*—H···*A*	*D*—H	H···*A*	*D*···*A*	*D*—H···*A*
C2—H2···O5^i^	0.93	2.56	3.442 (2)	159
C3—H3···O4^ii^	0.93	2.58	3.305 (2)	135
C5—H5···O2^iii^	0.93	2.46	3.132 (2)	129
C15—H15···O3^iv^	0.93	2.53	3.434 (2)	163
C19—H19A···O2	0.97	2.52	3.137 (2)	122

Symmetry codes: (i) −*x*+1, −*y*+1, −*z*+1; (ii) −*x*+2, −*y*, −*z*+1; (iii) *x*+1, *y*−1, *z*; (iv) −*x*+1, −*y*, −*z*+2.
